# Effect of Dietary Lipid Level on Growth Performance, Body Composition, and Physiometabolic Responses of Genetically Improved Farmed Tilapia (GIFT) Juveniles Reared in Inland Ground Saline Water

**DOI:** 10.1155/2022/5345479

**Published:** 2022-11-11

**Authors:** Mritunjoy Paul, Parimal Sardar, Narottam Prasad Sahu, Prasanta Jana, Ashutosh Dharmendra Deo, Vungurala Harikrishna, Tincy Varghese, Nazeema Shamna, Pankaj Kumar, Gopal Krishna

**Affiliations:** ^1^Fish Nutrition Biochemistry and Physiology Division, ICAR-Central Institute of Fisheries Education, Panch Marg, Off Yari Road, Versova, Mumbai 400 061, India; ^2^Department of Aquaculture, College of Fisheries Science, Gumla, Birsa Agricultural University, 835 207, Ranchi, India; ^3^ICAR-Central Institute of Fisheries Education, Rohtak Centre, Lahli, Haryana 124 411, India; ^4^Fish Genetics and Biotechnology Division, ICAR-Central Institute of Fisheries Education, Panch Marg, Off Yari Road, Versova, Mumbai 400 061, India

## Abstract

A 60-day feeding trial was carried out to determine the effect of dietary lipid levels on growth and physiometabolic responses to optimize the dietary lipid requirement for maximizing the growth of Genetically Improved FarmedTilapia (GIFT) juveniles reared in inland ground saline water (IGSW) of medium salinity (15 ppt). Formulation and preparation of seven heterocaloric (389.56-449.02 Kcal digestible energy/100 g), heterolipidic (40-160 g/kg), and isonitrogenous (410 g/kg crude protein) purified diets were done for conducting the feeding trial. Random distribution of 315 acclimatized fish (mean weight 1.90 ± 0.01 g) was made in seven experimental groups such as CL_4_ (40 g/kg lipid), CL_6_ (60 g/kg lipid), CL_8_ (80 g/kg lipid), CL_10_ (100 g/kg lipid), CL_12_ (120 g/kg lipid), CP_14_ (140 g/kg lipid), and CL_16_ (160 g/kg lipid) with 15 fish per triplicate tank (fish density, 0.21 kg/m^3^). Respective diets were used for feeding the fish at satiation level three times daily. Results indicated that weight gain percentage (WG%), specific growth rate (SGR), protein efficiency ratio, and protease activity significantly increased up to 100 g lipid/kg fed group, and then the values significantly decreased. Muscle ribonucleic acid (RNA) content and lipase activity were highest in 120 g/kg lipid-fed group. RNA/DNA (deoxyribonucleic acid) and serum high-density lipoproteins levels of 100 g/kg lipid-fed group were significantly higher than 140, and 160 g/kg lipid-fed groups. The lowest feed conversion ratio was found in the 100 g/kg lipid-fed group. The amylase activity was significantly higher in 40 and 60 g lipid/kg fed groups. The whole-body lipid level was increased with increasing the dietary lipid levels, whereas, there was no significant difference in whole-body moisture, crude protein, and crude ash contents of all groups. Highest serum glucose, total protein and albumin, and albumin to globulin ratio and lowest low-density lipoproteins level were found in 140 and 160 g/kg lipid-fed groups. Serum osmolality and osmoregulatory capacity did not vary significantly, whereas carnitine palmitoyltransferase-I and glucose-6-phosphate dehydrogenase showed an increased and decreased trend, respectively, with the increasing dietary lipid levels. According to second-order polynomial regression analysis based on WG% and SGR, the optimum dietary lipid for GIFT juveniles in IGSW of 15 ppt salinity was found to be 99.1 and 100.1 g/kg, respectively.

## 1. Introduction

Intensification of aquaculture leads to more dependence on quality feed as it accounts for around 50-60% of the total operational cost, and judicial use of nutrients in feed can easily sustain the growth of the aquaculture sector [1, 2]. Therefore, the most crucial consideration is an adequate nutritional information, especially the knowledge of the dietary nutrient requirements of species in relation to age and environmental conditions [3]. Dietary lipid is one of the crucial nutrients after protein to spare protein for energy supply, and thus contributes an important role in optimizing the dietary protein for the growth of living organisms [4]. Dietary lipid also plays an important role in providing essential fatty acids, phospholipid, and fat-soluble vitamins to fish [5, 6]. Fish prefer to catabolize dietary protein to satiate the physiometabolic energy needs than other nutrients [7]. Nevertheless, a diet with a higher amount of protein and lower levels of nonprotein energy sources accelerates ammonia production through preferential amino acid catabolism, resulting in the deterioration of the water quality and poor growth of fish [2, 8]. Therefore, during feed formulation, optimization of dietary protein to energy ratio (P : E) by increasing lipid content can spare protein for promoting the growth of fish with minimized nitrogenous output in the environment [1]. High dietary lipid levels can hamper the feed manufacturing process and reduce feed intake, thus resulting in the animal's poor growth performance [9]. Likewise, excess dietary lipid levels beyond the optimum level can cause abdominal fat deposition and fatty liver syndrome due to increased leptin levels [10], which affects the general wellbeing of the fish and the characteristic muscle quality [11]. So, lipid must be included in the fish diet at an optimum level to maximize protein utilization for better growth [4].

Lipid content of the diet can affect the digestibility of nutrients, activities of substrate-specific digestive enzymes, and the ribonucleic acid (RNA) to deoxyribonucleic acid (DNA) ratio in the muscle of the fish [12]. The gastrointestinal digestive enzyme activity is an important biochemical indicator of feeding activity [13]. The digestive enzyme activities of fish are positively correlated to the dietary nutrient digestion and absorption for growth [1] and are particularly regulated by specific substrates present in the fish's gut [14]. In fish, new tissue protein synthesis and deposition for growth are interlinked with muscle RNA levels corresponding to RNA to DNA ratio, and thus considered a suitable biomarker for evaluating the growth metrics of fish [15, 16].

Higher growth rate, tolerance of a wide range of salinity, efficient feed conversion efficiency, ease of spawning, resistance to diseases, and increased consumers preference have made tilapia as the world's second-largest farmed species after carps [17]. Unlike brackish, estuarine and marine water, inland ground saline water (IGSW) is deficient in potassium (K^+^) ion but high in calcium (Ca^2+^) and magnesium (Mg^2+^) ions [14, 18], which affects the physiological homeostasis and growth performance of fish [19]. Although growth rate is high in freshwater, Nile tilapia is least tolerant to salinity compared to other species of tilapia. However, it can tolerate a salinity up to 25 ppt [20], tilapia, especially the GIFT strain of Nile tilapia (*Oreochromis niloticus*), can be a suitable species for culturing in IGSW of 15 ppt as they have higher growth rate and similar salinity tolerance when compared to Nile tilapia [21]. However, tilapia can be cultured in IGSW without fortifying the K^+^ ion in IGSW [18, 22].

Different studies have reported that optimum dietary crude lipid for maximum growth of GIFT can be different under freshwater culture conditions in different age groups such as 85.6 g/kg for larvae [23], 93.4 g/kg for juveniles [24], and 75 g/kg for adult male [25]. Additionally, Hybrid tilapia [26] and GIFT [27] requires 120 and 92.2 g/kg dietary lipid, respectively, at the culture system of 10 and 8 ppt salinity. Reports on dietary lipid requirements of GIFT are available in freshwater and brackish water but limited for IGSW. Salinity difference is reported to influence the requirement of different energy nutrients [18]. Keeping the views mentioned in mind, the current study is aimed at optimizing the dietary lipid for juveniles of GIFT reared in IGSW of 15 ppt ambient salinity conditions with respect to growth, nutrient utilization, digestive enzyme activities, and muscle RNA/DNA ratio.

## 2. Materials and Methods

### 2.1. Fish Husbandry, Experimental Facilities, and Feeding Trial

The GIFT juveniles (mean weight 1.50 ± 0.01 g) were purchased and transported from Rajiv Gandhi Centre for Aquaculture (RGCA), India, in polythene bag containing oxygenated water to ICAR-Central Institute of Fisheries Education (ICAR-CIFE), Rohtak station, Haryana, India. Stocking of fish was made in a cemented tank (4 m × 2.5 m × 1 m; 12,000 L capacity), containing freshwater with the facility of continuous aeration. An underground pump collected IGSW of identical salinity, passed through a filtration unit (100 *μ*m), and kept into tanks (3 m × 2 m × 1.5 m, 9000 L capacity) for settlement. After two weeks, the IGSW was filled into storage tanks (1.06 m^2^ × 0.88 m, 950 L capacity) and used in the experiment. Fish were fed with a commercial diet (350 g/kg protein and 60 g/kg lipid, Avanti Feeds, India) on a satiation basis three times per day for two weeks acclimation. Then, the addition of IGSW was done with rising of 1 ppt per day until achieving the final salinity of 15 ppt, in which the fish were further acclimatized for three weeks with the same feeding schedule and continuous aeration facility.

After acclimatization, 315 fish (mean weight 1.90 ± 0.01 g) were arbitrarily dispersed in seven experimental groups such as CL_4_ (40 g lipid/kg), CL_6_ (60 g lipid/kg), CL_8_ (80 g lipid/kg), CL_10_ (100 g lipid/kg), CL_12_ (120 g lipid/kg), CP_14_ (140 g lipid/kg), and CL_16_ (160 g lipid/kg) in triplicates following completely randomized design (CRD). Fifteen fish were stocked (stocking density 0.21 kg/m^3^) in each circular tank (93 cm dia and 44 cm height, 325 L capacity with 225 L water volume) fitted with continuous aeration, and 12 h photoperiod was maintained. Respective diets were used for feeding the fish on a satiation basis three times per day (10.00, 14.00, and 18.00 h). At every fifteen days interval, the body weight was checked to judge the growth rate. Faeces from each tank were siphoned out prior to morning first feeding, and IGSW was replenished with an equal volume of siphoned water from the storage tank. About 20-25% experimental water from every tank was exchanged with IGSW of 15 ppt salinity from the storage facility at the interval of three days during the trial period. The animal experiment conducted were strictly adhered to the recommended guidelines for animal care and use after approval of Research Ethics Committee, ICAR-CIFE, Mumbai, India (University under Sec.  3 of University Grants Commission Act, and ISO 9001 : 2008 certified), while conducting the current study. The approval of the synopsis was obtained with no: FNT-PA7-04 from the university.

Everyday water quality parameters like pH, temperature, and salinity were estimated by pH meter (Aquafins Instruments, India), thermometer (Merck Millipore, Germany), and refractometer (Aquafins Instruments, India). Dissolved oxygen (DO), total alkalinity, total hardness, free carbon dioxide (CO_2_)_,_ nitrate-N, ammonia-N, nitrite-N, Ca^2+^, and Mg^2+^ ion concentrations were estimated following “APHA” procedures [28]. K^+^ concentration was estimated in a flame photometer (Essico Instruments, India). The analysis of the water quality parameters was done at two day's intervals.

The physicochemical parameters like pH, temperature, and salinity in the experimental groups were found within the range of 8.12 ± 0.11, 29.21 ± 0.24°C, and 15.45 ± 0.15 g/L, respectively. However, parameters like dissolved oxygen, total hardness, total alkalinity, Ca^2+^, Mg^2+^, K^+^, nitrite-N, ammonia-N, and nitrate-N were estimated in the range of 6.17 ± 0.09 mg/L, 2937.84 ± 13.29 mg/L, 262.45 ± 2.39 mg/L, 351.78 ± 3.17 mg/L, 505.65 ± 1.45 mg/L, 10.29 ± 0.97 mg/L, 0.002 ± 0.001 mg/L, 0.06 ± 0.03 mg/L, and 0.04 ± 0.02 mg/L, respectively. Free CO_2_ was not detected in water during 60 days experimental period.

### 2.2. Experimental Diet Formulation and Preparation

Ingredients were used for formulation (Table 1) and preparation of seven isonitrogenous (410 g/kg crude protein, CP) and heterolipidic (40-160 g/kg crude lipid, CL) purified experimental diets such as CL_4_ (40 g/kg lipid), CL_6_ (60 g/kg lipid), CL_8_ (80 g/kg lipid), CL_10_ (100 g/kg lipid), CL_12_ (120 g/kg lipid), CP_14_ (140 g/kg lipid), and CL_16_ (160 g/kg lipid). All the milled ingredients except oils, choline chloride, betaine, butylated hydroxytoluene (BHT), stay C (protected vitamin C), and vitamin-mineral mixture were mixed uniformly followed by addition of required quantity of water to form dough. Dough was then steam cooked for 25 min in a pressure cooker (121°C). After cooling, rest of the raw materials was added, thoroughly mixed and dough was prepared. Pellets (1 mm diameter) were then prepared by a pelletizer (S.B. Panchal & Co., India) and dried under air for overnight. On the very next day, the pellets were mechanically dried in the drier (42°C) till reaching around 90-100 g/kg moisture level. The pellet strands were properly crushed to adjust with mouth size of experimental fish followed by packaging in polythene bags, labelling, and storing at 4°C until used for feeding [29].

### 2.3. Growth Performance and Survival Rate

Following one-day feed restriction, the weight measurement of the whole fish was performed by electronic weighing balance at the beginning and after the completion of trial to calculate growth parameters as follows [30]:

(1) $$\begin{array}{c} \text{Weight gain }\left(\text{WG},\%\right)=\frac{\text{Final wet weight }\left(\text{g}\right)-\text{Initial wet weight }\left(\text{g}\right)}{\text{Initial wet weight }\left(\text{g}\right)}\times 100, \end{array}$$

(2) $$\begin{array}{c} \text{Specific growth rate }\left(\text{SGR},\frac{\%}{\text{day}}\right)=\frac{\left(\ln\text{of final wet weight}-\ln\text{of initial wet weight}\right)}{\text{Duration of feeding trial in days}}\times 100, \end{array}$$

(3) $$\begin{array}{c} \text{Feed conversion ratio }\left(\text{FCR}\right)=\frac{\text{Dry matter intake }\left(\text{g}\right)}{\text{Wet body weight gain }\left(\text{g}\right)}, \end{array}$$

(4) $$\begin{array}{c} \text{Protein efficiency ratio }\left(\text{PER}\right)=\frac{\text{Wet body weight gain }\left(\text{g}\right)}{\text{Protein intake on dry matter basis }\left(\text{g}\right)}, \end{array}$$

(5) $$\begin{array}{c} \text{Lipid efficiency ratio }\left(\text{LER}\right)=\frac{\text{Wet body weight gain }\left(\text{g}\right)}{\text{Lipid intake on dry matter basis }\left(\text{g}\right)}. \end{array}$$

After completion of the trial, live fish of every experimental tank was counted for calculation of survival as follows:

(6) $$\begin{array}{c} \text{Survival }\left(\%\right)=\frac{\text{Total number of live fish harvested at the end of feeding trial}}{\text{Total number of fish stocked at the commencement of feeding trial}}\times 100. \end{array}$$

### 2.4. Proximate Composition

Initially, whole 20 fish were pooled to one sample and after the end of 60 days trial period with overnight feed restriction, 15 fish from each treatment (5 from each tank) were pooled to one sample as for proximate analysis. Methods of AOAC [31] were employed for proximate analysis of whole-body and experimental diets. Samples were kept in a drier at 80°C till constant weight was achieved to determine the moisture content. The micro-Kjeldahl method (Kjeltech, Pelican Instrument, India) was followed for the estimation of the crude protein (CP), but lipid was estimated by solvent extraction (petroleum ether) method using soxhlet apparatus (SOCS plus, Pelican Instrument, India). Samples were incinerated by a muffle furnace (Wilser and Tetlow, Australia) (550°C for 6 h) for determining the crude ash (CA) content of samples. The crude fibre (CF) content analysis of fat-free samples was performed through acid and alkali digestion method in crude fibre assembly (Tulin Equipment, India) followed by burning digested sample in a muffle furnace at 550°C for 6 h. The subtraction method was employed to calculate NFE (nitrogen-free extract) of experimental diets and TC (total carbohydrate) of fish samples.

(7) $$\begin{array}{c} \text{NFE }\left(\frac{\text{g}}{\text{kg}}\right)=1000–\left\{\text{CP }\left(\frac{\text{g}}{\text{kg}}\right)+\text{EE }\left(\frac{\text{g}}{\text{kg}}\right)+\text{CF }\left(\frac{\text{g}}{\text{kg}}\right)+\text{CA }\left(\frac{\text{g}}{\text{kg}}\right)\right\}, \end{array}$$

(8) $$\begin{array}{c} \text{TC }\left(\%\right)=\left\{100–\left(\text{CP}\%+\text{EE}\%+\text{CA}\%\right)\right\}. \end{array}$$

Determination of gross energy (GE) of diet was performed in a Bomb Calorimeter (Changsu Instrument, China) assembly. Digestible energy (DE) [32] and protein to energy ratio (P : E) of diets were determined based on the following equations:

(9) $$\begin{array}{c} \text{DE }\left(\frac{\text{Kcal}}{100\text{g}}\right)=\left\{\left.\text{CP }\left(\frac{\text{g}}{100}\text{g}\right)\times 4\right)+\left.\text{ EE}\left(\frac{\text{g}}{100}\text{g}\right)\times 9\right)+\text{NFE }\left(\frac{\text{g}}{100}\text{g}\right)\times 4\right\}, \end{array}$$

(10) $$\begin{array}{c} \text{P}:\text{E }\left(\frac{\text{mg protein}}{\text{Kcal DE}}\right)=\frac{\text{CP}\left(\left(\text{g}/100\right)\text{g}\right)}{\left(\text{DE}\left(\text{Kcal}/100\text{g}\right)\times 1000\right)}. \end{array}$$

### 2.5. Assays of Digestive Enzymes

After trial completion, three fish were randomly collected from every experimental tank and anaesthetized by clove oil emulsion (Himedia Laboratories, India, 50 *μ*L/L) [18]. A small piece from the extreme anterior portion of intestine was then dissected out [33] and pooled to prepare the intestinal tissue homogenate under the iced condition to analyse the digestive enzyme activities.

#### 2.5.1. Tissue Homogenate Preparation

The collected intestinal tissue was immediately mixed with a chilled solution of sucrose (0.25 M), and a 5% tissue homogenate was prepared under iced condition using a homogenizer (Remi Equipment, India) coated with Teflon. Then, centrifugation (4°C) of the samples was performed for 10 min at 5000 rpm using a centrifuge (ThermoFisher Scientific, USA), and after collecting supernatants in cryo vials, the samples were stored at −20°C until used for digestive enzyme assays.

#### 2.5.2. Quantification of Tissue Protein

The protein content of the tissue homogenate was estimated by employing Lowry's method [34], and the obtained value was used to calculate digestive enzymes activities.

#### 2.5.3. Digestive Enzyme Assay

The activity of protease (millimole of tyrosine released/min/mg protein) was estimated as per Drapeau [35]. The activity of amylase (micromole of maltose released/min/mg protein) was analysed according to Rick and Stegbauer [36]. The titrimetric method of Cherry and Crandall [37] was followed to analyse lipase activity (unit/h/mg protein).

### 2.6. RNA and DNA Quantification and RNA-DNA Ratio

Muscle sample was carefully dissected out following the same procedure of intestinal digestive enzyme activities and immediately a 20% tissue homogenate was prepared. The quantification of muscle nucleic acids were done according to Schneider [38]. DNA was estimated by the diphenylamine method and RNA estimated by the orcinol. Briefly, 2.5 mL of cold 10% trichloroacetic acid (TCA) was added with 1 mL of 20% muscle homogenate and centrifuged at 5000 rpm for 10 min. The precipitate was washed by adding same volume of TCA. Then, precipitate was extracted twice with 5 mL of 95% ethanol by centrifugation at 5000 rpm for 10 min. Next, 2 mL 1 N potassium hydroxide (KOH) was added with lipoid compound free sample and incubated at 37°C for 20 h. After incubation, DNA and protein were precipitated by the addition of 0.4 mL 6 N hydrochloric acid (HCl) and 2 mL 5% TCA and centrifuged. After centrifugation, supernatant containing RNA is separated, and 0.2 mL supernatant was diluted by adding 1.5 mL distilled water. Then, diluted sample was heated by 1.5 mL orcinol reagent for 10 min and the absorbance was measured at 660 nm using a spectrophotometer (ThermoFisher Scientific, USA) for RNA content. On the other hand, sediment containing DNA was brought in to solution by heating with 2 mL 5% TCA. After cooling, 2 mL diphenylamine added with 1 mL solution and heated for 10 min in boiling water and the absorbance was measured at 600 nm for DNA. The following formulae were used for the calculation.

(11) $$\begin{array}{c} \text{DNA }\left(\mu \frac{\text{g}}{\text{mL}}\right)=\left(\frac{\text{OD at }600\text{ nm}}{0.019}\right), \end{array}$$

(12) $$\begin{array}{c} \text{RNA }\left(\mu \frac{\text{g}}{\text{mL}}\right)=\frac{\left[\left.\left(\text{OD at }660\text{ nm}+0.008\right)-\mu \text{g}\left(\text{DNA}/\text{mL}\right)\times 0.013\right)\right]}{0.116}, \end{array}$$

(13) $$\begin{array}{c} \text{RNA}-\text{DNA ratio}=\left\{\text{RNA}\frac{\mu \left(\text{g}/\text{mL}\right)}{\text{DNA}}\left(\mu \frac{\text{g}}{\text{mL}}\right)\right\}. \end{array}$$

### 2.7. Serum Biochemical Indices

#### 2.7.1. Collection of Serum

From each replicate tank, two fishes were sampled for drawing blood and subsequent serum preparation. For serum collection, the required volume of blood was drawn by puncturing the caudal vein using a hypodermal tuberculin syringe (DispoVAN India Ltd.) and immediately poured into eppendorf tube and kept undisturbed for 2 h under normal room temperature conditions. After proper clotting of the blood, tubes were subjected to centrifugation (7000 rpm for 12 min, 4°C) followed by the careful collection of straw-coloured serum and stored at -20°C until used for analysis.

#### 2.7.2. Analysis of Serum Biochemical Indices

Serum glucose, cholesterol, triglyceride, total protein, albumin, HDL, and LDL were assessed using a commercially available kit (ERBA Diagnostics, India). Globulin content (g/dL) was calculated by subtracting the total albumin value from the total protein value (g/dL), and the total albumin value (g/dL) was divided by total globulin value to compute the albumin to globulin ratio (A/G).

### 2.8. Serum Osmolality, Water Osmolality, and Osmoregulatory Capacity (OC)

Serum osmolality and water osmolality were analysed using an automated cryoscopic osmometer (Vapro™, GMbH, Germany). Osmoregulatory capacity (OC) was estimated by subtracting water osmolality from serum osmolality [39]. Osmolality and OC were expressed in mOsmol/kg.

### 2.9. Hepatic Lipid Metabolic Enzymes

Liver sample was collected in the same manner like intestinal digestive enzymes and a 5% tissue homogenate was prepared and stored in −20°C until used for hepatic lipid metabolic enzymes assay.

#### 2.9.1. Carnitine palmitoyl CoA I (CPT-I)

The liver CPT-I activity (mM DTNB, 5,5-dithiobis-2-nitrobenzoic acid converted/min/mg protein) was analysed according to Zheng true [40]. A mixture was prepared by mixing 116 mM tris buffer solution (pH 8.0), tissue homogenate, and 1 mM palmitoyl CoA. The reaction was initiated after adding 1.2 mM carnitine solution (pH 8.0), and the optical density (OD) value (412 nm) was recorded for 3 min.

#### 2.9.2. Glucose-6-Phosphate Dehydrogenase (G6PDH)

The liver G6PDH activity (mM phosphorus released/min/mg protein) was assayed following the method of De Moss [41]. A mixture was prepared by mixing 0.1 M tris buffer (pH 7.8), 2.7 mM nicotinamide adenine dinucleotide phosphate (NADP), 0.1 M magnesium chloride (MgCl_2_), and tissue homogenate. After adding 0.2 M glucose-6-phosphate as substrate, the reaction was initiated, and the OD value (340 nm) was recorded for 3 min.

### 2.10. . Statistical Analysis

All the results were expressed as the mean ± SE (*n* = 3 tank per treatment). Data evaluation for the assumption, *η* including normality and homogeneity of variance using Shapiro-Wilk and Levene's tests, respectively, and there was no violation of these assumptions (*p* > 0.05). For statistical comparison of data among the diets, one-way analysis of variance (ANOVA) was employed in SPSS software (Version 22.0). Post hoc analysis and Duncan's multiple range tests were set to observe the significant differences among the means (*p* < 0.05). A second-degree polynomial model [42] was employed for weight gain and specific growth rate values for optimizing dietary lipid requirement of GIFT juveniles in rearing condition of IGSW at 15 ppt salinity.

## 3. Results

### 3.1. . Proximate Composition of Experimental Diets

Lipid levels were 41.1, 61.6, 81.7, 101.0, 121.5, 141.2, and 161.8 g/kg in the experimental diets of CL_4_, CL_6_, CL_8_, CL_10_, CL_12_, CP_14_, and CL_16_, respectively, and crude protein (CP), gross energy (GE), digestible energy (DE), and protein to energy ratio (P : E) values ranged between 408.2-409.3 g/kg, 487.92-552.24 Kcal/100 g, 389.56-449.02 Kcal/100 g, and 91.15-104.78 mg protein/Kcal DE, respectively (Table 1).

### 3.2. Growth Performance and Survivability

**Figure 1:**
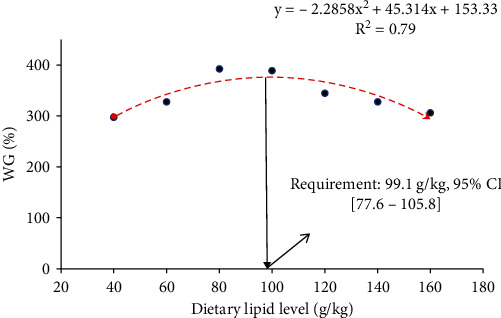
In relation to weight gain percentage (WG%), second order polynomial regression model indicates the optimum dietary crude lipid requirement (g/kg) of GIFT juveniles reared in IGSW of 15 ppt for 60 days (ANOVA: *F*_6,14_ = 55.62, *p* = <_0.001, confidence interval, CI for mean at 95% level = 324.31-358.32).

**Figure 2:**
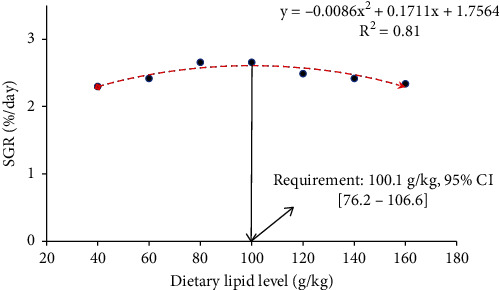
In relation to specific growth rate (SGR, %/day), second order polynomial regression model indicates the optimum dietary crude lipid requirement (g/kg) of GIFT juveniles reared in IGSW of 15 ppt for 60 days (ANOVA: *F*_6,14_ = 59.37, *p* = <_0.001, confidence interval, CI for mean at 95% level = 2.41-2.53).

The growth performance, including WG%, SGR, FCR, and PER of GIFT juveniles fed diets with graded lipid levels are shown in Table 2. There was a significant increment (DMRT: *p* < 0.05) of final body weight (FBW) (ANOVA: *F*_6,14_ = 56.76, *p* < _0.001, confidence interval for mean at 95% level = 8.03-8.63, and the effect size of one–way ANOVA, *η*^2^ = 0.96) and WG% (ANOVA: *F*_6,14_ = 55.62, *p* < _0.001, confidence interval for mean at 95% level = 324.31-358.32, and the effect size of one–way ANOVA, *η*^2^ = 0.96), and SGR (ANOVA: *F*_6,14_ = 59.37, *p* < _0.001, confidence interval for mean at 95% level = 2.41-2.53, and the effect size of one–way ANOVA, *η*^2^ = 0.96) in relation to enhancing dietary lipid levels up to 100 g/kg and then gradually decreases with the further increase in the dietary lipid levels. With the opposite trend, FCR (ANOVA: *F*_6,14_ = 65.49, *p* < _0.001, confidence interval for mean at 95% level = 1.61-1.74, and the effect size of one–way ANOVA, *η*^2^ = 0.97) decreased significantly (DMRT: *p* < 0.05) with the increasing the dietary lipid levels up to 100 g/kg, and further increment of dietary lipid levels caused the increasing trend of FCR. PER increased significantly (DMRT: *p* < 0.05) up to 100 g lipid/kg fed group (ANOVA: *F*_6,14_ = 55.75, *p* < _0.001, confidence interval for mean at 95% level = 1.42-1.52 and the effect size of one–way ANOVA, *η*^2^ = 0.96) and then gradually decreased. Significantly linear decreasing trend (DMRT: *p* < 0.05) of lipid efficiency ration (LER) (ANOVA: *F*_6,14_ = 408.82, *p* < _0.001, confidence interval for mean at 95% level = 5.66-8.51, and the effect size of one–way ANOVA, *η*^2^ = 0.99) was found with the increasing dietary lipid level up to maximum. Based on WG% and SGR, second-order polynomial regression analysis revealed that the optimum dietary crude lipid requirement of GIFT juveniles under the rearing condition of IGSW of 15 ppt salinity was 99.1 g/kg (Figure 1) and 100.1 g/kg (Figure 2) with DE value of around 400 Kcal/100 g and corresponding P :  E values of 98.45 and 97.46 mg protein/Kcal DE, respectively. All dietary groups showed 100% survival rate (Table 2).

### 3.3. Proximate Composition of the Whole Body of Fish

Whole-body initial moisture, crude protein, lipid, total ash, and total carbohydrate contents of fish were 764.33, 137.46, 39.71, 36.69, and 22.09 g/kg, respectively. No significant variation (*p* > 0.05) was found for whole-body final moisture (ANOVA: *F*_6,14_ = 2.09, *p* = 0.12, confidence interval for mean at 95% level = 749.70-755.93, and the effect size of one–way ANOVA, *η*^2^ = 0.47), crude protein (ANOVA: F_6,14_ = 1.33, *p* = 0.31, confidence interval for mean at 95% level = 142.65-146.22, and the effect size of one–way ANOVA, *η*^2^ = 0.36), total ash (ANOVA: *F*_6,14_ = 1.27, *p* = 0.33, confidence interval for mean at 95% *level* = 25.06-26.78, and the effect size of one–way ANOVA, *η*^2^ = 0.35), and total carbohydrate (ANOVA: *F*_6,14_ = 1.36, *p* = 0.30, confidence interval for mean at 95% level = 11.28-17.93, and the effect size of one–way ANOVA, *η*^2^ = 0.37), but whole-body lipid content of fish significantly varied (ANOVA: *F*_6,14_ = 4.03, *p* = 0.02, confidence interval for mean at 95% level = 58.16-66.30, and the effect size of one–way ANOVA, *η*^2^ = 0.63) in relation to dietary lipid levels (Table 3). The fish of CL_16_ exhibited significantly the higher (DMRT: *p* < 0.05) whole-body lipid content than the fish of CL_4_ group, although lipid content of CL_16_ was not significantly different (DMRT: *p* > 0.05) from CL_8_, CL_10_, CL_12_, and CL_14_ groups and CL_4_ group from CL_6_, CL_8_, and CL_10_ groups. The increasing trend of whole-body lipid levels was linked with increasing levels of dietary lipid.

### 3.4. Digestive Enzymes

Intestinal protease, amylase, and lipase activities showed significant variation (DMRT: *p* < 0.05) in relation to graded levels of dietary lipid (Table 4). The intestinal protease activity of CL_8_ and CL_10_ groups were significantly higher (ANOVA: *F*_6,14_ = 11.07, *p* < _0.001, confidence interval for mean at 95% level = 0.25-0.28, and the effect size of one–way ANOVA, *η*^2^ = 0.83) than that of other dietary groups. Lipase activity of CL_8_, CL_10_,and CL_12_ groups were higher (ANOVA: *F*_6,14_ = 24.26, *p* < _0.001, confidence interval for mean at 95% level = 0.33-0.43, and the effect size of one–way ANOVA, *η*^2^ = 0.91) than other dietary groups. Whereas, the amylase activity was significantly (ANOVA: *F*_6,14_ = 8.69, *p* < _0.001, confidence interval for mean at 95% level = 10.32-11.32, and the effect size of one–way ANOVA, *η*^2^ = 0.79) decreased in higher lipid-fed groups (CL_8_, CL_10_, CL_12_, CL_14_, and CL_16_) in comparison to lower lipid-fed groups (CL_4_ and CL_6_).

### 3.5. RNA, DNA, and RNA-DNA Ratio

Effect of graded levels of dietary lipid on muscle DNA concentration, RNA concentration, and the value of RNA to DNA ratio (RNA/DNA) are presented in Table 5. DNA content of muscle did not vary significantly (ANOVA: *F*_6,14_ = 0.05, *p* = 0.10, confidence interval for mean at 95% level = 20.98-21.51, and the effect size of one–way ANOVA, *η*^2^ = 0.02) among the dietary groups, whereas RNA content of CL_10_ and CL_12_ was similar (DMRT: *p* > 0.05) to that of CL_8_ and significantly higher (ANOVA: *F*_6,14_ = 13.15, *p* < _0.001, confidence interval for mean at 95% level = 8.05-8.53, and the effect size of one–way ANOVA, *η*^2^ = 0.85) than the other groups. However, the value of RNA to DNA ratio of lower lipid-fed groups (CL_4_ and CL_6_) was significantly lower (ANOVA: *F*_6,14_ = 4.67, *p* = 0.01, confidence interval for mean at 95% level = 0.38-0.40, and the effect size of one–way ANOVA, *η*^2^ = 0.67) than that of CL_8_, CL_10_, and CL_12_ groups, although RNA/DNA of CL_10_ group was similar (DMRT: *p* > 0.05) to that of CL_8_ and CL_12_ groups and significantly higher (DMRT: *p* < 0.05) than that of CL_14_ and CL_16_ groups. No significant variation (DMRT: *p* > 0.05) was found among CL_8_, CL_12,_ CL_14_, and CL_16_ groups.

### 3.6. Serum Glucose and Lipid Profiles

Feeding of the graded levels of dietary lipid significantly influenced (DMRT: *p* < 0.05) the serum glucose (GLU), total cholesterol (T-CHO), high-density lipoproteins (HDL), and low-density lipoproteins (LDL) levels in fish, but serum triacylglycerol (TAG) levels did not vary significantly (DMRT: *p* > 0.05) among the dietary groups (Table 6). Serum GLU contents of the CL_10_ group was significantly higher (*p* < 0.05) than that of CL_4_, CL_6_, and CL_8_ groups and lower than CL_12_, CL_14_, and CL_16_ groups. Serum GLU levels was similar (*p* > 0.05) among CL_4_, CL_6_, and CL_8_ groups and CL_12_, CL_14_, and CL_16_ groups, but higher lipid-fed groups showed significantly higher (ANOVA: *F*_6,14_ = 11.44, *p* < _0.001, confidence interval for mean at 95% level = 5.64-6.31, and the effect size of one–way ANOVA, *η*^2^ = 0.83) serum GLU levels than the lower lipid-fed groups. The serum T-CHO levels of the CL_10_ group was similar (DMRT: *p* > 0.05) to CL_8_ and CL_12_ groups but significantly higher (ANOVA: *F*_6,14_ = 5.58, *p* = 0.01, confidence interval for mean at 95% level = 2.32-2.45, and the effect size of one–way ANOVA, *η*^2^ = 0.71) than other dietary groups. However, serum HDL levels of CL_8_ and CL_10_ groups were similar (DMRT: *p* > 0.05) to the CL_12_ group and significantly higher (ANOVA: *F*_6,14_ = 5.13, *p* = 0.01, confidence interval for mean at 95% level = 1.35-1.46, and the effect size of one–way ANOVA, *η*^2^ = 0.69) than the other dietary groups. On the other hand, serum LDL levels of CL_16_ group was similar (DMRT: *p* > 0.05) to CL_14_ group and significantly lower (ANOVA: *F*_6,14_ = 3.51, *p* = 0.03, confidence interval for mean at 95% level = 0.87-0.96, and the effect size of one–way ANOVA, *η*^2^ = 0.60) than that of other dietary groups, although CL_14_ group showed similar (*p* > 0.05) serum LDL contents with lower lipid-fed groups.

### 3.7. Serum Protein Profile

Feeding varying levels of dietary lipid significantly (DMRT: *p* < 0.05) influenced the serum protein profile except for globulin (DMRT: *p* > 0.05) of the treatment groups (Table 6). The serum total protein level of the CL_10_ group and albumin levels of the CL_8_, CL_10_, and CL_12_ groups were significantly higher (DMRT: *p* < 0.05) than that of the lower lipid-fed groups and lower than that of the higher lipid-fed groups. However, higher lipid-fed groups showed significantly more serum total protein (ANOVA: *F*_6,14_ = 31.98, *p* < _0.001, confidence interval for mean at 95% level = 3.48-3.62, and the effect size of one–way ANOVA, *η*^2^ = 0.93)and albumin (ANOVA: *F*_6,14_ = 27.75, *p* < _0.001, confidence interval for mean at 95% level = 1.32-1.51, and the effect size of one–way ANOVA, *η*^2^ = 0.92) levels than that of lower lipid-fed groups. However, serum albumin to globulin ratio (A/G) significantly (ANOVA: *F*_6,14_ = 9.64, *p* = 0.01, confidence interval for mean at 95% level = 0.61-0.72, and the effect size of one–way ANOVA, *η*^2^ = 0.80) increased with an increase in dietary lipid levels.

### 3.8. Serum Osmolality, Water Osmolality, and Osmoregulatorycapacity (OC)

Serum osmolality, water osmolality, and osmoregulatory capacity did not show any significant difference (DMRT: *p* > 0.05) with different dietary lipids in experimental diets (Table 7).

### 3.9. Lipid Metabolic Enzymes Activity

The hepatic CPT-I and G6PDH activities were significantly (DMRT: *p* < 0.05) different among the treatments (Table 8), and the hepatic CPT-I activities increased significantly (ANOVA: *F*_6,14_ = 4.28, *p* = 0.01, confidence interval for mean at 95% level = 0.16-0.18, and the effect size of one–way ANOVA, *η*^2^ = 0.65) with increasing dietary lipid levels up to CL_12_ group. However, further increase in dietary lipid levels from the CL_12_ group reduced the CPT-I activity. But, the CL_14_ and CL_16_ groups were nonsignificant among themselves. Furthermore, the G6PDH activity in the liver decreased significantly (ANOVA: *F*_6,14_ = 5.52, *p* = 0.01, confidence interval for mean at 95% level = 0.14-0.15, and the effect size of one–way ANOVA, *η*^2^ = 0.70) with the increase in dietary lipid levels, and the highest value was recorded in the 40 g/kg lipid-fed group (CL_4_). The lower values were observed in CL_14_ and CL_16_ groups.

## 4. Discussion

Growth, the muscle hyperplasia of animals, including fish, is mainly regulated via dietary and environmental conditions [43]. Dietary lipid provides energy to the animals, thus sparing amino acids for energy production and making it available for fish growth. Therefore, optimum dietary lipid level reduces the dietary protein requirement with optimizing the protein to energy ratio (P : E), in which almost all dietary protein derived amino acids undergo synthesis and deposition of muscle protein to causes the maximum growth of fish with reduced environmental pollution in terms of nitrogenous waste [3]. Moreover, less dietary protein reduces the feed cost to make the aquaculture production economic. In the present study, the WG%, SGR, and PER of GIFT juveniles enhanced significantly with elevating dietary lipid levels up to 100 g/kg with lowest FCR and beyond that it caused significant reduction of growth and protein utilization with enhanced FCR. This finding clearly indicates that feeding excess lipid beyond the optimum level for GIFT reared in IGSW of 15 ppt salinity was not beneficial in terms of growth, which might be due to the reason that excess lipid with imbalanced P : E probably reduces the protein digestibility and availability of amino acids for production and deposition of somatic tissue protein leading reduced growth of fish [44]. Moreover, excess dietary lipid probably causes the metabolic burden to fish with the outcome of reduced growth [45]. In accordance with the present finding, Mohanta true [46] and Sivaramakrishnan true [11] reported growth retardation in pangas and silver barb, respectively, due to feeding of excess dietary lipid with imbalanced P : E. On the other hand, feeding of low dietary lipid, as found in the present study, also causes reduction of growth of fish may be owing to less availability of lipid as nonprotein energy source, which probably directs the dietary protein derived amino acids towards catabolism for energy production at the cost of body protein synthesis [47]. Therefore, optimum protein, lipid, and P : E of aquafeed maximize growth of fish with minimizing feed cost, less degradation of water quality through nitrogenous discharges, and profitable aquaculture production. In our study, based upon the second degree polynomial analysis of WG% and SGR,the optimum dietary lipid requirement of GIFT juveniles reared in IGSW of 15 ppt salinity was found to be 99.1 and 100.1 g/kg, respectively, at the dietary protein level of 410 g/kg. In the line of the present finding, Mohammadi true [27] reported that 90 g/kg dietary lipid could be optimum at 360 g/kg dietary protein level for all male tilapia in brackish water of 8 ppt salinity. In the present study, the salinity (15 ppt) and ionic composition of inland saline water was different from that of brackish water used in the study by Mohammadi true [27], which resulted in the higher lipid requirement. However, optimum dietary lipid level for Nile tilapia [48], juvenile hybrid tilapia [26], adult GIFT [25], and larval GIFT [23] in freshwater condition was reported to be 91.0, 120.3, 87.9, and 85.6 g/kg, respectively. Therefore, the present finding is the first report of dietary lipid requirement for juveniles of GIFT reared under the IGSW of 15 ppt ambient salinity condition.

FCR is the indicator of feed utilization in relation to the growth of animals, including fish that depends upon the feed quality, condition of fish, and environmental factors [14, 43]. In the present study, FCR showed the opposite trend of fish growth in relation to dietary lipid levels. A similar observation was also made by Mohammadi true [27] in Nile tilapia (all male) in the brackish water of 8 ppt salinity. PER value indicates the utilization capacity of protein by animals, including fish. Thus, in our study, higher PER and growth in 100 g/kg lipid fed GIFT juveniles indicates maximum utilization of dietary protein-derived amino acids for production and deposition of somatic tissue protein. A similar observation was demonstrated by Qiang true [23] for GIFT and Mohammadi true [27] for all male tilapia in freshwater and brackish water conditions, respectively. In this study, complete survival of the animal in the entire experiment group suggests that neither water quality parameters nor the experimental diets were fatal to the GIFT juveniles in IGSW because water quality parameters in the present study were found within the recommended limits for inland saline water fish culture [14, 18].

Whole body composition indicates the nutritional quality of feed and nutrient utilization efficiency, flesh quality, and wellbeing of fish [49]. In this study, increased body lipid was found in higher lipid-fed groups. In agreement with the present finding, Peres and Oliva-Teles [50], Yildirim-Aksoy true [51], Mohanta true [46], and Sivaramakrishnan true [11] demonstrated the positive correlation between dietary lipid levels and whole-body lipid content of fish indicating excess dietary lipid could cause the deposition of body fat irrespective of culture condition. A similar finding was also demonstrated in Nile tilapia [52], adult GIFT [25], and larval GIFT [23] in freshwater culture conditions. On the other hand, no significant changes were found in other components of the proximate composition of fish. In contrast to the present finding, other studies in several fishes [11, 23, 26, 46] demonstrated the higher ash content of body was due to feeding of higher dietary lipid. However, Tian true [25] found higher whole-body ash content in the lower dietary lipid-fed group. But, the exact reason for these variable observations remains unclear. Decreased whole-body moisture content in rohu [53], silver barb [46], adult GIFT [25], and larval GIFT [23] of higher dietary lipid-fed group also opposed the present observation.

Digestive enzyme activities are positively linked with fish growth, probably through enhancing nutrient digestibility and utilization [54]. In our study, protease activity of 80 and 100 g/kg lipid-fed groups and lipase activity of 80, 100, and 120 g/kg lipid-fed groups were significantly higher, whereas amylase activity was significantly lower in fish fed with dietary lipid levels beyond 60 g/kg. The protease and lipase activity in relation to dietary lipid level indicates that 80-100 g/kg dietary lipid probably could be optimally utilized to spare dietary protein for energy production, and 410 g/kg dietary protein at this lipid level could be properly digested, and derived amino acids were almost available for somatic tissue synthesis leading to maximum growth [55] as proved in this study. Gangadhara true [56], Mohanta true [46], and Sivaramakrishnan true [11] demonstrated similar findings in rohu (*Labeo rohita)*, silver barb (*Puntius gonionotus*), and sutchi catfish (*Pangasiodon hypophthalmus*), respectively. Lower lipase activity in the lower dietary lipid-fed groups might be due to the availability of limited substrate in the gastrointestinal tract, and probably higher dietary carbohydrates could suppress the protease activity of these groups. Lower protease and lipase activity in very high lipid-fed groups indicates that excess dietary lipid probably could suppress the activity of proteolytic and lipolytic enzymes [6, 57]. On the other hand, the increased and decreased amylase activity in lower and higher lipid-fed groups of this study might be due to higher and lower levels of digestible carbohydrates intake [6].

Muscle RNA content and RNA-DNA ratio has a positive correlation with the tissue protein synthesis and accretion for the growth of fish, thus used as a growth marker [1]. In this study, higher muscle RNA concentration and RNA/DNA in 80, 100, and 120 g/kg lipid-fed groups were positively correlated with higher growth of fish, probably due to the protein-sparing effect of dietary lipid at these levels to influence more body protein synthesis and accretion. In corroboration with this finding, Mohanta true [46] and Sivaramakrishnan true [11] observed that RNA/DNA ratio increased in the *P. gonionotus* and *P. hypophthalmus* with increasing the dietary lipid up to the optimum level to correlate the enhanced growth. In our study, muscle DNA content showed a nonsignificant variation among the treatment groups and was well supported by the observations of Mohanta et al. [46] and Kumar true [1] in *P.gonionotus* and *L.rohita*, respectively.

Serum glucose level is an important stress biomarker in fish [58]. In our study, serum GLU level increased with an increase in dietary lipid levels. This finding indicates that the fishes were stressed due to feeding a high lipid diet associated with higher metabolic burden and energy needs [59]. Wang true [24] also concluded a similar report in GIFT juveniles fed with varying dietary lipid levels reared in freshwater conditions. In fish, serum triglyceride level reflects the magnitude of lipid catabolism in the body for energy production [6]. Thus, in our study, the higher serum TAG values in very high-dietary lipid-fed groups probably could indicate the lower lipid utilization due to metabolic burden [25]. Similar observations were also reported by Wang true [24] in GIFT (*O. niloticus*), Jin true [60] in grass carp (*Ctenopharyngodon idella*), and Nayak true [61] in silver barb (*P. gonionotous*) to corroborate the present finding.

High-density lipoproteins (HDL) carry excess cholesterol to the liver, where it can be either used or excreted through bile [62]. Low-density lipoproteins (LDL) carries cholesterol to the living cells, but very high-serum LDL is indicator of poor health status of animals, including fish [63]. Serum T-CHO, HDL, and LDL levels can also be regulated via fish diet [64, 65]. In the current study, serum T-CHO and HDL concentrations enhanced with dietary lipid levels up to 100 g/kg and then decreased with increased dietary lipid content. This decreasing trend of serum T-CHO and HDL levels in a high-fat diet beyond the optimum level may indicate poor liver function and chances of parenchymal liver disease [66]. In agreement with the present finding, Tian true [25] reported increased serum T-CHO and HDL levels in tilapia, Kikuchi true [67] and Wang true [24] in tiger puffer and GIFT, and Guo true [9] in largemouth bass (*Micropterus salmoides*) due to feeding of optimum level of dietary lipid. However, in our study, serum LDL exhibited a significantly decreasing trend with increasing dietary lipid and excessive decrease of serum LDL in very high lipid-fed groups might indicate the liver dysfunction [68]. However, a contrasting report by Deng true [6] described that dietary lipid levels did not influence the serum T-CHO, HDL and LDL concentration in Asian red-tailed catfish, *Hemibagrus wyckioides*.

Serum protein profile can be used as the indicator of nutritional, metabolic, and health status of fish [13, 69]. In this study, the higher lipid-fed group showed significantly higher serum total protein concentrations than the lower lipid-fed groups. This increment of serum total protein might be owing to the transportation of excess dietary lipid as lipoprotein in the blood of higher lipid-fed groups. Lim true [52] and Yildirim-Aksoy true [70] reported similar observations in Nile tilapia and channel catfish, respectively. Among the serum proteins, globulin and albumin levels indicate the general well–being of fish [71, 72]. Albumin content of serum exhibited significantly increased values with enhancing dietary lipid up to a maximum level, which was similar to the finding of Wang true [73] in Crucian carp. In the present experiment, the highest albumin and the lowest globulin content were found in the CL_14_ and CL_16_ groups, suggesting probable damage and malfunctioning of liver cells of GIFT in response to higher dietary lipid levels.

There were no significant differences in serum and water osmolality among the treatment, and the fact was that salinity did not affect serum osmolality of GIFT. The results indicated that GIFT adapted to the high water salinity of IGSW at 15 ppt during the culture periods. Moreover, the serum osmolality of fishes remains the same among the different treatment groups as osmolality is more a function of water salinity, which was constant in the experiment [2]. According to Verdegem true [74] and Jana true [3], the diet composition does not affect serum osmolality.

Carnitine palmitoyltransferase-I(CPT-I) is the main regulatory enzyme in mitochondrial fatty acid oxidation [75]. The hepatic CPT-I activity was significantly elevated with increasing dietary lipid levels in many fishes, and it has been reported that feeding high-fat diets will increase CPT-I expression compared with low-fat diets in large yellow croakers [76] and grass carp [77]. In the present study, the higher lipid levels beyond 120 g/kg suppressed the hepatic CPT-I activity might be due to excess intake of fat. This result is in corroboration with the results reported by Guo true [9], who observed a reduced CPT-I activity with very high-dietary fat in largemouth bass. Similar observations were also reported in blunt snout bream, where the activity and expression of CPT-I were significantly downregulated in fish fed the high-fat diet [78].

Glucose-6-phosphate dehydrogenase (G6PDH) is a key enzyme in catalyzing nicotinamide adenine dinucleotide phosphate hydrogen (NADPH) production, essential for hepatic fatty acid biosynthesis [79]. In the present study, the G6PDH activities of the liver in relation to increasing dietary lipid exhibited a reducing trend from lower lipid to high-lipid diets. Higher activity of liver G6PDH in fish fed the low-lipid content diet than other lipid-containing diets suggest that the lipid biosynthesis is active in this group. Similar results were observed in hybrid tilapia [26] and juvenile cobia [80]. Additionally, dietary carbohydrate supply is also known to affect enzyme activities [80]. It has been confirmed in many fish species that high-carbohydrate diets stimulate lipogenesis and are conversely suppressed by high-dietary lipid [80–82]. However, the decrease in this enzyme activity with higher-lipid levels reveals that the high-dietary lipid depressed lipogenic enzyme activities in the liver of tilapia, as suggested by Shimeno true [83]. In the present experiment, the higher dietary carbohydrate content might be higher G6PDH activity of dietary lipid in fish fed with a 40 g lipid/kg diet.

## 5. Conclusion

Knowing the optimum dietarylipidin relation to growth, body composition, and physiobiochemical responses, a nutritionally balanced, cost-effective, and environmentally friendly diet can be formulated for the fish. Accordingly, based on WG% and SGR, second-order polynomial regression analysis optimized the dietary lipid requirement of GIFT juveniles reared condition of IGSW (15 ppt salinity) at the level of 99.1and 100.1 g/kg with the corresponding P :  E value of 98.45and 97.46 mg protein/Kcal DE, respectively. Thus, the optimum dietary crude lipid requirement of GIFT juveniles in IGSW of 15 ppt salinity could range between 99.1 and 100.1 g/kg to maximize fish growth. This finding will help to develop nutritionally balanced diet for culturing GIFT in IGSW.

## Figures and Tables

**Table 1:** Formulation and proximate composition of the experimental diets fed to GIFT juveniles cultured in IGSW of 15 ppt for 60 days.

Ingredients (g/kg)	Diets (experimental groups)^1^						
	CL_4_	CL_6_	CL_8_	CL_10_	CL_12_	CL_14_	CL_16_
Casein^2^	388	388	388	388	388	388	388
Gelatin^2^	97	97	97	97	97	97	97
Dextrin^2^	100	100	100	100	100	100	100
Starch^2^	279.5	259.5	239.5	219.5	199.5	179.5	159.5
Cellulose^2^	50	50	50	50	50	50	50
Fish oil^3^	20	30	40	50	60	70	80
Sunflower oil^4^	20	30	40	50	60	70	80
Vit-min mix^5^	15	15	15	15	15	15	15
CMC^6^	20	20	20	20	20	20	20
BHT^7^	0.2	0.2	0.2	0.2	0.2	0.2	0.2
Choline chloride^2^	2.5	2.5	2.5	2.5	2.5	2.5	2.5
Betaine^2^	7.5	7.5	7.5	7.5	7.5	7.5	7.5
Stay C^8^	0.3	0.3	0.3	0.3	0.3	0.3	0.3
Total	1000	1000	1000	1000	1000	1000	1000

Proximate composition (on g/kg dry weight basis)							
Moisture	86.3	84.2	82.8	85.2	84.6	85.2	85.3
Crude protein	408.2	408.8	408.5	408.9	408.3	409.2	409.3
Ether extract	41.1	61.6	81.7	101.0	121.5	141.2	161.8
Crude fibre	51.7	52.0	51.5	52.6	52.4	52.2	52.8
Crude ash	25.7	26.1	26.3	25.8	26.4	26.7	26.9
NFE^9^	473.3	451.4	432.0	411.7	391.4	370.8	349.2
GE^10^ (kcal/100 g)	487.92	498.64	509.36	520.08	530.80	541.52	552.24
DE^11^ (kcal/100 g)	389.56	399.55	409.70	419.11	429.22	439.04	449.02
P: E^12^ (mg protein/kcal DE)	104.78	102.32	99.71	97.56	95.13	93.20	91.15

^1^CL_4_ (40 g/kg dietary crude lipid), CL_6_ (60 g/kg dietary crude lipid), CL_8_ (80 g/kg dietary crude lipid), CL_10_ (100 g/kg dietary crude lipid), CL_12_ (120 g/kg dietary crude lipid), CL_14_ (140 g/kg dietary crude lipid), and CL_16_ (160 g/kg dietary crude lipid). ^2^Ingredients procured from Himedia Pvt. Ltd., India; ^3^procured from Seacod Oil by Sanofi India Ltd., India; ^4^purchased from local retail shop, India. ^5^Composition of vitamin-mineral mixture (quantity/kg): vitamin A, 550,000 IU; vitamin D3, 110,000 IU; vitamin B2, 2000 mg; vitamin E, 750 mg; vitamin K, 1000 mg; vitamin B6, 1000 mg; vitamin B12, 6 mcg; calcium pantothenate, 2500 mg; nicotinamide, 10 g; choline chloride,150 g; Mn, 27,000 mg; I, 1000 mg; Fe, 7500 mg; Zn, 5000 mg; Cu, 2000 mg; Co, 450 L-lysine, 10 g; DL-methionine, 10 g; selenium 50 ppm. ^6^Carboxymethyl cellulose, purchased Himedia Pvt. Ltd., India; ^7^butylated hydroxytoluene, purchased Himedia Pvt. Ltd., India; ^8^ROVIMIX® STAY-C®35 purchased from DSM in Animal Nutrition and Health, India; ^9^nitrogen free extract; ^10^gross energy; ^11^digestible energy; ^12^protein to energy ratio.

**Table 2:** Growth, nutrient utilization, and survival of GIFT juveniles cultured in IGSW of 15 ppt and fed with experimental diets for 60 days.

Experimental groups^1^	IBW^2^	FBW^3^	WG%^4^	SGR^5^ (%/day)	FCR^6^	PER^7^	LER^8^
CL_4_	1.90 ± 0.01	7.54 ± 0.05^a^	297.56 ± 4.23^a^	2.30 ± 0.02^a^	1.85 ± 0.02^e^	1.33 ± 0.01^a^	13.18 ± 0.04^g^
CL_6_	1.90 ± 0.01	8.13 ± 0.09^b^	327.49 ± 5.56^b^	2.42 ± 0.02^b^	1.80 ± 0.02^de^	1.36 ± 0.02^a^	9.04 ± 0.23^f^
CL_8_	1.88 ± 0.02	9.24 ± 0.07^c^	392.25 ± 4.00^d^	2.66 ± 0.01^d^	1.62 ± 0.02^c^	1.51 ± 0.02^b^	7.57 ± 0.10^e^
CL_10_	1.87 ± 0.01	9.26 ± 0.17^c^	394.08 ± 9.98^d^	2.66 ± 0.03^d^	1.48 ± 0.02^a^	1.65 ± 0.03^d^	6.68 ± 0.14^d^
CL_12_	1.88 ± 0.03	8.32 ± 0.05^b^	344.32 ± 3.46^c^	2.49 ± 0.01^c^	1.56 ± 0.01^d^	1.57 ± 0.01^c^	5.31 ± 0.31^c^
CL_14_	1.89 ± 0.01	8.07 ± 0.08^b^	327.48 ± 3.25^b^	2.42 ± 0.01^b^	1.63 ± 0.02^c^	1.50 ± 0.02^b^	4.36 ± 0.05^b^
CL_16_	1.90 ± 0.01	7.73 ± 0.07^a^	306.04 ± 0.37^a^	2.34 ± 0.00^a^	1.79 ± 0.01^d^	1.36 ± 0.01^a^	3.45 ± 0.05^a^
*p* value	0.092	<0.001	<_0.001	<_0.001	<_0.001	<_0.001	<0.001

Data are expressed as mean ± SE (*n* = 3 tanks from each treatment). Mean values in the same column with different superscripts differ significantly (*p* < 0.05). ^1^CL_4_-CL_16_, 40-160 g/kg dietary crude lipid. ^2^IBW: initial body weight; ^3^FBW: final body weight; ^4^WG%: weight gain percentage; ^5^SGR: specific growth rate; ^6^FCR: feed conversion ratio; ^7^PER: protein efficiency ratio; ^8^LER: lipid efficiency ratio.

**Table 3:** Whole body proximate composition (on g/kg wet weight basis) of GIFT juveniles cultured in IGSW of 15 ppt and fed with experimental diets for 60 days.

Experimental groups^1^	Moisture	Crude protein	Lipid	Total ash	Total carbohydrate
IBC^2^	764.33 ± 1.16	137.46 ± 0.58	39.71 ± 0.27	36.69 ± 0.39	22.09 ± 0.48
FBC^3^					
CL_4_	760.11 ± 1.28	140.93 ± 0.71	49.54 ± 3.14^a^	27.48 ± 1.21	21.94 ± 2.85
CL_6_	759.00 ± 0.11	141.28 ± 0.43	58.35 ± 4.98^ab^	26.62 ± 1.61	14.76 ± 7.05
CL_8_	753.49 ± 7.91	143.89 ± 4.79	60.28 ± 4.00^abc^	25.51 ± 0.68	16.83 ± 2.18
CL_10_	750.80 ± 1.37	147.10 ± 2.08	61.15 ± 5.06^abc^	23.86 ± 0.54	17.09 ± 5.46
CL_12_	750.00 ± 3.01	146.56 ± 1.82	64.37 ± 1.27^bc^	25.22 ± 0.69	13.85 ± 1.33
CL_14_	748.59 ± 1.72	146.01 ± 0.66	70.47 ± 2.39^bc^	26.16 ± 0.62	8.78 ± 1.84
CL_16_	747.74 ± 2.06	145.27 ± 0.96	71.40 ± 3.76^c^	26.60 ± 1.41	8.98 ± 3.87
*p* value	0.120	0.301	0.015	0.323	0.298

Data are expressed as mean ± SE (*n* = 3 pooled fish per tank). Mean values in the same column with different superscripts differ significantly (*p* < 0.05). ^1^CL_4_-CL_16_, 40-160 g/kg dietary crude lipid. ^2^IBC: initial body composition; ^3^FBC: final body composition.

**Table 4:** Digestive enzymes activities in the intestine of GIFT juveniles cultured in IGSW of 15 ppt and fed with experimental diets for 60 days.

Experimental groups^1^	Protease (millimole of tyrosine released/min/mg protein)	Amylase (micromole maltose released/min/mg protein)	Lipase (unit/h/mg protein)
CL_4_	0.21 ± 0.01^a^	12.56 ± 0.5^b^	0.28 ± 0.01^a^
CL_6_	0.24 ± 0.01^b^	11.99 ± 0.21^b^	0.30 ± 0.04^a^
CL_8_	0.30 ± 0.00^c^	10.66 ± 0.32^a^	0.48 ± 0.01^b^
CL_10_	0.31 ± 0.01^c^	10.27 ± 0.25^a^	0.54 ± 0.03^b^
CL_12_	0.26 ± 0.01^b^	10.20 ± 0.51^a^	0.47 ± 0.02^b^
CL_14_	0.26 ± 0.02^b^	10.05 ± 0.04^b^	0.34 ± 0.03^a^
CL_16_	0.25 ± 0.01^b^	10.00 ± 0.37^a^	0.27 ± 0.01^a^
*p* value	<_0.001	<_0.001	<_0.001

Data are expressed as mean ± SE (*n* = 3 pooled fish per tank). Mean values in the same column with different superscripts differ significantly (*p* < 0.05). ^1^CL_4_-CL_16_, 40-160 g/kg dietary crude lipid.

**Table 5:** DNA and RNA contents and RNA-DNA ratio in the muscle of GIFT juveniles cultured in IGSW of 15 ppt and fed with experimental diets for 60 days.

Experimental groups^1^	DNA^2^ (*μ*g/mL)	RNA^3^ (*μ*g/mL)	RNA/DNA^4^
CL_4_	21.12 ± 0.27	7.65 ± 0.11^a^	0.36 ± 0.00^a^
CL_6_	21.16 ± 0.42	7.79 ± 0.06^a^	0.37 ± 0.01^a^
CL_8_	21.33 ± 0.35	8.63 ± 0.14^cd^	0.41 ± 0.01^bc^
CL_10_	21.37 ± 0.40	9.01 ± 0.25^d^	0.42 ± 0.02^c^
CL_12_	21.3 ± 0.37	8.72 ± 0.07^d^	0.41 ± 0.00^bc^
CL_14_	21.23 ± 0.59	8.24 ± 0.16^bc^	0.39 ± 0.02^ab^
CL_16_	21.19 ± 0.34	8.00 ± 0.09^ab^	0.38 ± 0.01^ab^
*p* value	0.999	<_0.001	0.008

Data are expressed as mean ± SE (*n* = 3 pooled fish per tank). Mean values in the same column with different superscripts differ significantly (*p* < 0.05). ^1^CL_4_-CL_16_, 40-160 g/kg dietary crude lipid. ^2^DNA: deoxyribonucleic acid; ^3^RNA: ribonucleic acid; ^4^RNA/DNA, RNA to DNA ratio.

**Table 6:** Serum glucose, lipid, and protein profile of GIFT juveniles cultured in IGSW of 15 ppt and fed with experimental diets for 60 days.

Experimental groups^1^	GLU^2^ (mmol/L)	TAG^3^ (mmol/L)	T-CHO^4^ (mmol/L)	HDL^5^ (mmol/L)	LDL^6^ (mmol/L)	Total protein (g/dL)	Albumin (g/dL)	Globulin (g/dL)	A/G^7^
CL_4_	5.20 ± 0.13^a^	1.79 ± 0.16	2.26 ± 0.05^a^	1.29 ± 0.03^a^	1.01 ± 0.04^b^	3.35 ± 0.03^a^	1.11 ± 0.04^a^	2.25 ± 0.04	0.49 ± 0.03^a^
CL_6_	5.26 ± 0.08^a^	1.87 ± 0.18	2.34 ± 0.06^abc^	1.31 ± 0.07^a^	0.98 ± 0.05^b^	3.38 ± 0.03^a^	1.21 ± 0.04^a^	2.17 ± 0.06	0.56 ± 0.04^ab^
CL_8_	5.60 ± 0.14^a^	1.95 ± 0.20	2.50 ± 0.06^cd^	1.52 ± 0.04^c^	0.93 ± 0.03^b^	3.44 ± 0.02^a^	1.38 ± 0.04^b^	2.06 ± 0.06	0.67 ± 0.04^bc^
CL_10_	5.71 ± 0.07^ab^	2.03 ± 0.10	2.59 ± 0.04^d^	1.54 ± 0.05^a^	0.91 ± 0.05^b^	3.58 ± 0.03^b^	1.42 ± 0.04^b^	2.16 ± 0.07	0.66 ± 0.04^bc^
CL_12_	6.32 ± 0.38^bc^	2.13 ± 0.13	2.45 ± 0.04^bcd^	1.47 ± 0.04^bc^	0.91 ± 0.03^b^	3.68 ± 0.03^c^	1.50 ± 0.05^b^	2.18 ± 0.07	0.69 ± 0.04^cd^
CL_14_	6.82 ± 0.27^c^	2.19 ± 0.19	2.31 ± 0.08^ab^	1.37 ± 0.03^ab^	0.88 ± 0.05^ab^	3.68 ± 0.02^c^	1.63 ± 0.04^c^	2.05 ± 0.06	0.80 ± 0.04^d^
CL_16_	6.89 ± 0.21^c^	2.20 ± 0.08	2.24 ± 0.04^a^	1.33 ± 0.02^ab^	0.77 ± 0.03^a^	3.73 ± 0.03^c^	1.65 ± 0.03^c^	2.08 ± 0.04	0.79 ± 0.03^d^
*p* value	<_0.001	0.434	0.004	0.006	0.025	<_0.001	<_0.001	0.207	<_0.001

Data are expressed as mean ± SE (*n* = 3 pooled fish per tank). Mean values in the same column with different superscripts differ significantly (*p* < 0.05). ^1^CL_4_-CL_16_, 40-160 g/kg dietary crude lipid. ^2^GLU: glucose; ^3^TAG: triacylglycerol; ^4^T-CHO: total cholesterol; ^5^HDL: high-density lipoprotein cholesterol; ^6^LDL: low-density lipoprotein cholesterol; ^7^A/G: albumin to globulin ratio.

**Table 7:** Serum osmolality, water osmolality, and osmoregulatory capacity (OC) of GIFT juveniles cultured in IGSW of 15 ppt and fed with experimental diets for 60 days.

Experimental groups^1^	Serum osmolality (mOsmol/kg)	Water osmolality (mOsmol/kg)	Osmoregulatory capacity (mOsmol/kg)
CL_4_	349.67 ± 1.33	246.33 ± 2.96	103.33 ± 2.96
CL_6_	348.67 ± 2.03	247.00 ± 1.73	101.67 ± 3.33
CL_8_	346.67 ± 2.03	248.67 ± 2.60	98.00 ± 4.00
CL_10_	345.67 ± 1.67	247.67 ± 2.40	98.00 ± 4.04
CL_12_	347.67 ± 2.73	247.33 ± 2.03	100.33 ± 3.84
CL_14_	347.00 ± 1.73	246.67 ± 1.76	100.33 ± 3.18
CL_16_	346.67 ± 3.28	248.33 ± 2.40	98.33 ± 4.10
*p* value	0.880	0.989	0.922

Data are expressed as mean ± SE (*n* = 3 pooled fish per tank). Mean values in the same column with different superscripts differ significantly (*p* < 0.05). ^1^CL_4_-CL_16_, 4-16% dietary crude lipid.

**Table 8:** Hepatic lipid metabolic enzymes activity of GIFT juveniles cultured in IGSW of 15 ppt and fed with experimental diets for 60 days.

Experimental groups^1^	CPT-I^2^	G6PDH^3^
CL_4_	0.15 ± 0.01^a^	0.16 ± 0.02^c^
CL_6_	0.16 ± 0.02^a^	0.15 ± 0.01^b^
CL_8_	0.17 ± 0.01^b^	0.15 ± 0.02^b^
CL_10_	0.18 ± 0.01^c^	0.14 ± 0.03^ab^
CL_12_	0.19 ± 0.01^d^	0.14 ± 0.01^ab^
CL_14_	0.18 ± 0.02^c^	0.13 ± 0.01^a^
CL_16_	0.18 ± 0.01^c^	0.13 ± 0.02^a^
*p* value	0.012	0.004

Data are expressed as mean ± SE (*n* = 3 pooled fish per tank). Mean values in the same column with different superscripts differ significantly (*p* < 0.05). ^1^CL_4_-CL_16_, 4-16% dietary crude lipid. ^2^CPT-I: Carnitine palmitoyltransferase-I, mM DTNB converted/min/mg protein. ^3^G6PDH, Glucose-6-phosphate dehydrogenase, mM phosphorus released/min/mg protein.

## Data Availability

The data that support the findings of this study are available from the corresponding author upon reasonable request.
